# ACT001 Alleviates chronic kidney injury induced by a high-fat diet in mice through the GPR43/AMPK pathway

**DOI:** 10.1186/s12944-023-01949-2

**Published:** 2023-11-18

**Authors:** Yibing Zhou, Ze Chen, Hui Zhou, Bin Niu, Jing Liu, Yinglun Li, Yuqiang Mi, Ping Li

**Affiliations:** 1 Clinical School of the Second People’s Hospital, Tianjin Medical University, Tianjin, China; 2 Department of Hepatology, Tianjin Second People’s Hospital, Tianjin, China; 3 Tianjin Research Institute of Liver Disease, Tianjin, China

**Keywords:** ORG, ACT001, SCFA, GPR43, AMPK

## Abstract

**Background:**

Roughly 10 -15% of global populace suffer from Chronic Kidney Disease(CKD). A major secondary disease that can progress to end-stage renal disease (ESRD) is obesity-associated kidney disease (ORG). Although clinical management strategies are currently available, morbidity and mortality rates are increasing. Thus, new solutions are needed. Intestinal permeability, systemic inflammation, and aberrant intestinal metabolites have all been linked to ORG.

**Purpose:**

ACT001 has anti-inflammatory, redox-regulatory and antitumour activities. The current study was designed to examine how ACT001 affects ORG and analyze the fundamental processes.

**Methods:**

A high-fat diet (HFD) was used to generate ORG in female C57BL/6 J mice.

ORG mice were divided into three groups at random: HFD, HFD + ACT001, HFD + polyphosphocholine (PPC). To assess renal and colonic damage, periodic acid-Schiff (PAS) and hematoxylin–eosin (HE) staining were used. Following that, renal inflammation, oxidative stress, lipid deposition, colonic inflammation, and intestinal permeability were evaluated by protein blotting, polymerase chain reaction (PCR), immunohistochemistry, and immunofluorescence staining. Lastly, the SCFAs content was assessed by gas chromatographymass spectrometry.

**Results:**

Mice in the HFD group displayed more severe albuminuria, glomerular hypertrophy, renal oxidative damage, inflammation, and lipid accumulation than mice with the normal diet (ND) group, as well as lower levels of intestinal SCFA valproic acid, colonic inflammation, and tight junction protein downregulation. ACT001 treatment restores the content of valproic acid in intestinal SCFAs, promotes the binding of SCFAs to renal GPR43, activates the AMPK signalling pathway. Therefore, it promotes the Nrf2-Keap1 signalling pathway and inhibits the NF-κB signalling pathway. SCFAs, additionally, augment colonic GPR43 concentrations, diminishing NLRP3 inflammasome expression and restoring ZO-1 and occludin protein levels.

**Conclusion:**

This study is the first to look at ACT001's potential as a treatment for obesity-related kidney disease. Regulating GPR43 and AMPK signalling pathways, By controlling the GPR43 and AMPK signalling pathways, ACT001 improves colitis and the intestinal mucosal barrier, decreases renal lipid deposition, and suppresses inflammation and oxidative stress in the kidneys. According to this study, ACT001 could be a viable ORG therapy option.

## Introduction

Nearly 15% of individuals worldwide are afflicted by Chronic Kidney Disease (CKD) [1]. In particular, in line with the global obesity epidemic, obesity-associated kidney disease (ORG) is becoming more common [2]. Epidemiological studies indicate that 10.8% of Chinese people have CKD. Unavoidably, this will result in significant societal hardship [3]. Currently, ORG still lacks specific therapeutic drugs. However, as the pathogenesis of ORG continues to be studied, targeted medicines that can enhance renal metabolism and suppress inflammatory pathways have gradually replaced traditional weight reduction and metabolic regulation as the main forms of ORG therapy [4].

Pathological alterations in the kidneys are brought on by key synergistic variables, including chronic inflammation and aberrant lipid levels [5]. Chronic inflammation was found to induce lipogenesis in nonadipose tissues, resulting in ectopic lipid deposition in mouse organs and causing tissue lipotoxicity [6]. Tissue lipotoxicity induces mitochondrial dysfunction and promotes oxidative and endoplasmic reticulum stress [7]. In chronic hyperlipidaemia, excessive lipid accumulation occurs in the kidneys and lipotoxicity drives pro-inflammatory response activation, fibrosis and apoptosis, resulting in cell damage and renal dysfunction [8].

Recently, recognition of the gut-renal axis has grown. Short-chain fatty acids (SCFAs) and their target receptors are among the physiological processes that the gut microbiome is crucial in controlling [9]. According to studies, SCFAs have a crucial role in energy metabolism by influencing how lipids, glucose, and cholesterol are metabolized as well as improving insulin sensitivity [10]. SCFAs are effective agonists of GPR43 [11]. Under dysbiosis conditions, the decreased synthesis of SCFAs results in the dysregulation of lipid homeostasis and the activation of GPR43 and AMPK [12]. Under typical conditions, SCFAs, as the colon epithelial cells' main energy source, are essential in sustaining intestinal epithelial cell morphology and operation as well as preserving the intestinal mucosal barrier's integrity [13]. When the intestinal barrier is structurally altered and functionally reduced, intestinal metabolites move through the weaken intestinal barrier and arrive in the submucosa. This induces generalized inflammation, which in turn aggravates the course of renal disease [14].

The American Food and Drug Administration (FDA) designated ACT001 as an orphan drug [15]. Derived from micheliolide(MCL), its anti-inflammatory, redox-modulating, and antitumor activities have all been confirmed [16]. The dimethylamino Michael adduct of MCL is known as DMAMCL, and the fumarate salt version of DMAMCL known as ACT001 is usually employed in vivo [17]. Because of its increased potency, longer-lasting release, and higher plasma stability, ACT001 has a higher oral bioavailability and greater therapeutic potential [18]. Interleukin-6 (IL-6) in individuals with idiopathic pulmonary fibrosis have been demonstrated to be reduced with ACT001. It also prevent the deposition of fibronectin caused by transforming growth factor β1 (TGF-β1). In non-alcoholic steatohepatitis (NASH), ACT001 can lower inflammation, oxidative damage, and steatosis [19, 20]. Therefore, ACT001 might affect GPR43 and AMPK activity by regulating SCFA content, which might alleviate ORG.

The therapeutic benefits of ACT001 on ORG were investigated in this research employing a HFD-induced ORG model in mice. After treatment with ACT001, pathological alterations in renal and colon tissues were found, relevant biochemical parameters were measured to assess the alleviating effect of ACT001 on ORG, and relative levels and types of SCFAs in mouse faeces were measured by GC‒MS to understand ACT001’s infiuence on intestinal SCFAs. Analyzing the extent of cytokine expression linked to inflammation, fibrosis, and lipid metabolism, research has found that ACT001 might increase GPR43 levels through valeric acid, promote lipid metabolism, inhibit inflammation and oxidative stress by mediating the AMPK signalling pathway. This is the first examination of ACT001’s therapeutic impact on the kidneys of high-fat mice. ACT001 has the potential to provide a novel treatment option to patients suffering from ORG.

## Materials and methods

### Materials

ACT001 was supplied by Accendatech Co., Ltd. (Tianjin, China, lot C10668-11).

Polyphosphocholine (PPC) was purchased from Sanofi Pharmaceuticals Ltd. (Beijing, China, lot ABJD465B).

### Animals

**Fig. 1:**
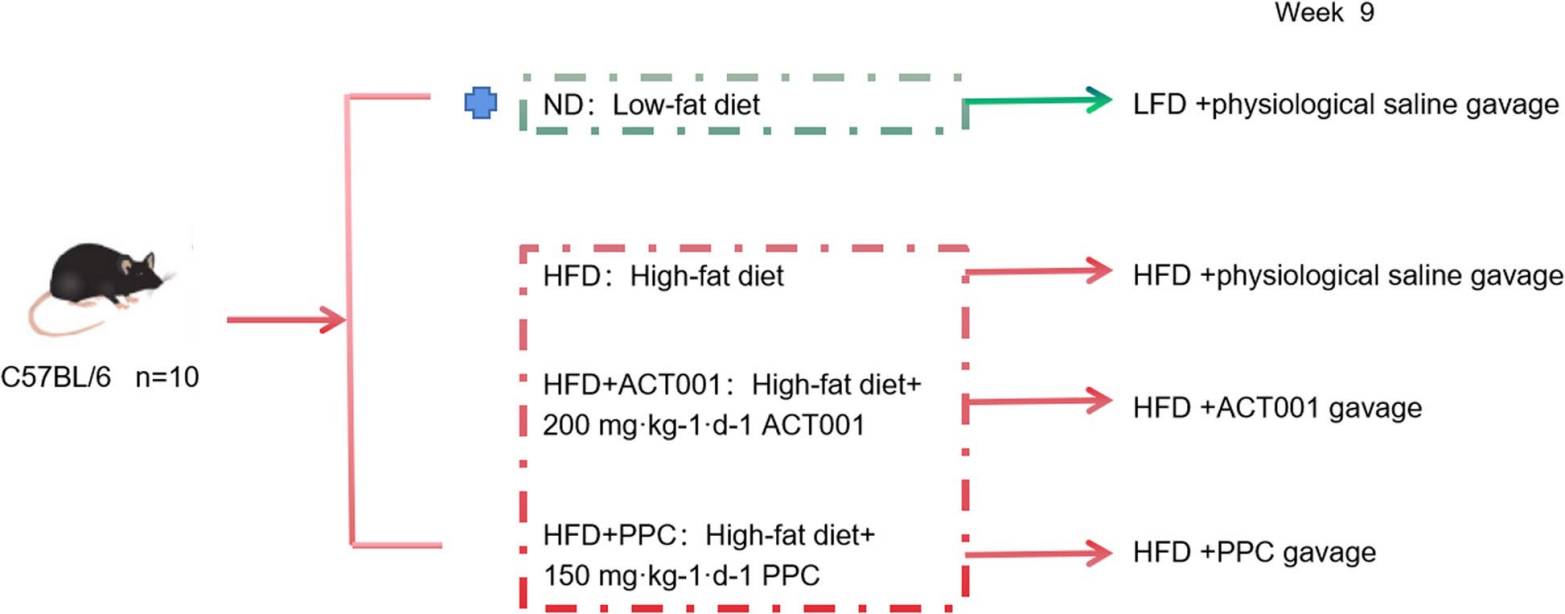
The outlook of the animal experiment

Beijing Huafukang Biotechnology Co., Ltd. provided C57BL/6 J female mice (20 ± 2 g, 6–8 weeks old) (certificate number SCXK (Beijing) 2019–0008). Forty C57BL/6 J female mice, 8 weeks old and weighing 20 ± 2 g, were selected. After a seven-day period of adaptation, 4 groups of mice were randomly assigned (10 per group): ND, HFD, HFD + ACT001 (200 mg/kg/d), HFD + PPC (150 mg/kg/d). The HFD group received an HFD containing 60% fat whereas the ND group received a low-fat diet (LFD) containing 10% fat (D12492, D12450J, Xiao Shu You Tai (Beijing) Biotechnology Co., Ltd, production licence number SCXK (Beijing) 2018–0006). The specific dietary composition is shown in Tables 1 and 2. Dissolving the drug in physiological saline for gavage, a dose was determined based on the animal's body mass. Figure 1 displays the design of the experiment conducted with animals. At week 20, all animals were euthanized. Urine/faecal, kidney and colon samples were collected.

**Table 1:** Physical composition of feed

Ingredient	Low fat feed (kcal)	High fat feed (kcal)
Casein, 30 Mesh	800	800
L-Cystine	12	12
Corn Starch	2024.8	0
Maltodextrin 10	500	500
Sucrose	275.2	275.2
Cellulose, BW200	0	0
Soybean Oil	225	225
Lard*	180	2205
Mineral Mix S10026	0	0
DiCalcium Phosphate	0	0
Calcium Carbonate Potassium	0	0
Citrate, 1 H2O	0	0
Vitamin Mix V10001	0	0
Choline Bitartrate	0	0
FD&C Yellow Dye #5	0	-
FD&C Blue Dye #1	0	0
Total	4075	4075

**Table 2:** Chemical composition of feed

Ingredien	Low fat feed (kcal)	High fat feed (kcal)
Protein	20%	20%
Carbohydrate	70%	20%
Fat	10%	60%

### Glucose tolerance test (IPGTT) and insulin tolerance test (IPITT)

After a period of 8–12 h of fasting, injection of 1 g/kg glucose solution was conducted to assess their glucose tolerence; blood was obtained at 0, 0.25, 0.5, 1 and 2 h post injection for real-time monitoring, and the area beneath each blood glucose curve(AUC)was calculated.

After the glucose tolerance test had been completed, a fasting of mice for 6–8 h and an injection of 0.75 U/kg human rapid-acting insulin was conducted to assess their resistance to insulin; blood glucose changes were then monitored at intervals of 0, 0.25, 0.5, 1 and 2 h post-injection, with AUC calculated as part of this process. After the experiment, iodophor was used for local disinfection to prevent infection.

### ELISA

Using the ELISA kit instructions(Nanjing Jiancheng and SEA124Mu), concentrations of TC, TG, and TGF-β in kidney tissue homogenate were ascertained, as well as trace albumin and creatinine levels in urine.

### Histopathology

Tissues were fixed for 72 h in 10% formalin-saline, gradient dealcoholized with xylene, eluted with water and embedded in paraffin. Slices of paraffin blocks were made at a thickness of 5 m, cleared with xylene, and later eluted in a reverse gradient using ethanol and water. Haematoxylin and eosin (HE) and periodic acid-Schiff (PAS) were used to stain the sections. All images were obtained using a pathology slide scanner (C13220-01, Japan).

### Frozen section preparation and oil red O staining

Wash 3 μm frozen-kidney sections in distilled water after fixing in 10% formalin for 5 min. Soak 100% 1,2-propanediol and wait another five min. Subsequently, the sections were stained with oil red O for 9 min at 60 °C. Finally, 85% 1,2-propanediol was added to the section after it had been washed with distilled water. Mayer's haematoxylin was then employed to stain the sections for 3 min before they were washed again in distilled water and installed in the aqueous solution. All images were obtained using a pathology slide scanner (C13220-01, Japan).

### Immunohistochemistry

After deparaffinizing the specimens with xylene and gradient ethanol, they were washed three times every 2 min in sterile PBS before being fixed with normal goat serum at 60 °C for 3 min. Subsequently, primary antibodies against INOS (BA0362), GPR43 (13536R), ZO-1 (ab276131), occludin (ab216327) and claudin (ab180158) were incubated overnight at 30 °C on the slices.After washing with phosphate buffered saline 3 times, slices were treated with secondary antibodies that were horseradish peroxidase (HRP)-labelled for 3 min. Then, 3,80 diaminobenzidine (Maxim# DAB40333) was served as a substrate for visualization of positive staining. Finally, a 3DHistech (Pannoramic MIDI, Hungary) was used to take the pictures.

### Immunofluorescence

With 3% BSA mounting solution, paraffin slices were sealed after being fixed with EDTA antigen repair buffer. Sequential additions of primary and secondary antibodies against ROS (D7008) and F4/80 (70076S). Anti-fluorescence quenching sealant was used to seal the nuclei after DAPI labeling before observation under a fluorescence microscope. Finally, images were acquired using 3DHistoch (Pannoramic MIDI, Hungary).

### SCFAs analysis

Twenty milligrams of faecal samples were precisely measured and put into a 2 mL EP tube. A milliliter of phosphoric acid solution (0.5% v/v) was put into the EP tube, along with a steel ball. Faecal samples underwent uniform grinding, a 10-min vortex, and a 5-min ultrasonic process. The mixture was centrifuged at 12,000 rpm for 10 min at 4℃. The supernatant was transferred to a 1.5 mL centrifuge tube containing 0.1 mL. Then add 0.5 mL of MTBE (containing internal standard) solution, vortexed for 3 min, and heated using ultrasonic energy for 5 min. The mixture was then centrifuged for 10 min at 4 ℃ using a 12,000 rpm speed. After that, the produced supernatant was gathered and used for gas chromatographymass spectrometry examination utilizing an Agilent 7890B-7000D. A chromatographic column (DB-FFAP 30 m × 0.25 mm × 0.25 μm) combined with 7000 D mass spectrometry was used. The film thickness was measured by gas chromatography–mass spectrometry, with helium serving as the carrier gas at a flow rate of 1.2 mL per min. A separate injection process was implemented on 2 μL samples.The oven temperature was maintained at 90 °C for 1 min, increased to 100 °C at 25 °C/min, increased to 150 °C at 20 °C/min, maintained for 0.6 min, increased to 200 °C at 25 °C/min and run for 3 min. Utilizing a multiple reaction monitoring mode, all samples were examined. The transmission line temperature was 230 °C, while the fuel injector's intake temperature was 200 °C.

### Quantitative real-time polymerase chain reaction (qRT‒PCR)

QRT‒PCR was employed to isolate and analyze total RNA, with primers crafted to assess the renal gene content of interleukin-6 (IL6) and tumour necrosis factor alpha (TNFa) (Table 3). The reaction system was set up in accordance with the usage instructions, and the following conditions were used for PCR amplification: 95 °C for 30 s for predenaturation; 95 °C for 15 s and 60 °C for 30 s cycled 40 times. Using GAPDH as a control, relative expression was measured using 2-ΔΔCt method.

**Table 3:** The sequences of the primers for RT-qPCR

Name	Primer sequence (5’-3’)
TNFa-F	CAGAAAGCATGATCCGCGAC
TNFa-R	TTGAGAAGATGATCTGAGTGTGAG
IL-6-F	CTTGGACTGATGCTGGTGA
IL-6-R	TTGGGAGTGGTATCCTCTGTGA

### Western blot

Protein lysis buffer was used to lyse the protein in the renal cortex, which was then quantified using a BCA detection kit(Solarbio #PC0020). Subsequently, sodium dodecyl sulfate‒polyacrylamide gel electrophoresis was employed to electrophorease the samples and they were transferred onto PVDF membranes. The primary antibody of choice was probed at 4 °C overnight on these membranes. Primary antibodies against AMPK (5831 T), p-AMPK (AP1002), ACC (3676 T), p-ACC (11818 T), ACL (ab40793), HMG CR (DF6518), p-p65 (ab76302), IKKβ (AF3010), NLRP3 (ab263899), NRF2 (DF7519), and Keap1 (ab227828) were used.

At room temperature, HRP-conjugated secondary antibody incubation was conducted for 2 h. Subsequently, the membranes were washed and inspected with an ECL kit (Tanon # 180–501) under a fluorescence chemiluminescence gel imaging system (# 3300 Mini). ImageJ was used to analyse all protein bands.

### Statistical analysis

SPSS 26.0 and GraphPad Prism 8.0 were used for the statistical analysis and graph processing, respectively. Univariate Analysis of Variance (ANOVA) was employed to compare groups, while Mann's' Whitney test was used to contrast nonparametric data between them; a *P* value lower than 0.05 signifying statistical significance.

## Results

### ACT001 may alleviate obesity and metabolic syndrome caused by long-term HFD feeding

**Fig. 2:**
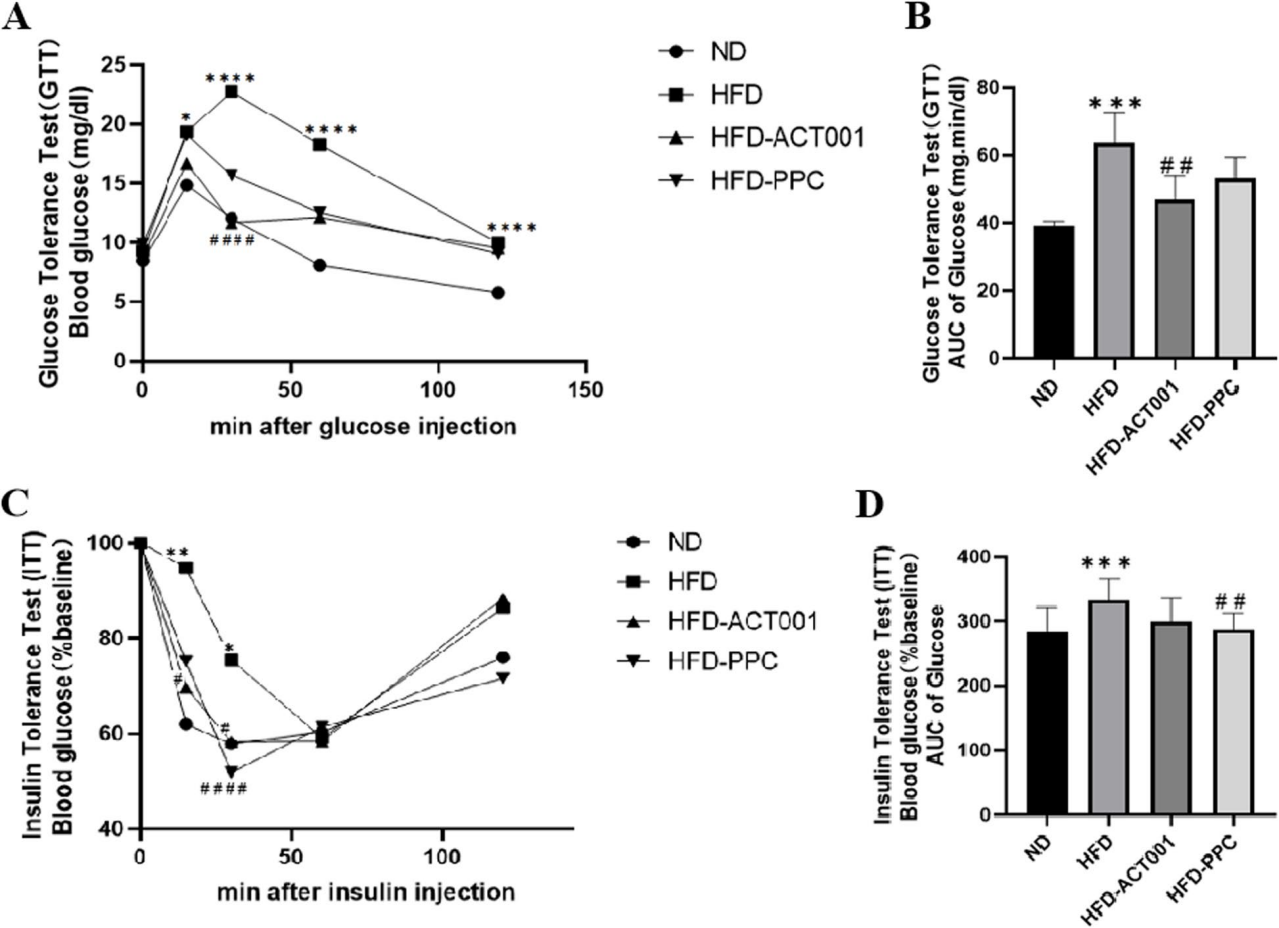
ACT001 may alleviate obesity and metabolic syndrome caused by long-term HFD feeding. (**A**) GTT and (**B**) AUC curse (**C**) ITT and (**D**) AUC curves *P < 0.05, **P < 0.01, ***P < 0.001, ****P < 0.0001 vs. ND; #*P* < 0.05, ##*P* < 0.01, ###*P* < 0.001, ####*P* < 0.0001 vs HFD

Previous research has indicated that long-term HFD-fed mice have larger body weights than the ND mice. The mice's weight did decrease following therapy with ACT001 or PPC. The GTT experiment's findings showed that, in comparison to the ND group mice, HFD group mice experienced a swift and significant rise in blood glucose levels, with the decline in glucose being relatively sluggish, suggesting a considerable decrease in glucose tolerance.Glucose tolerance improved after treatment with ACT001 or PPC (Fig. 2A-B). The ITT experiment revealed that, in comparison to the ND group, the HFD group displayed a slower and diminished decrease of blood glucose levels, implying considerable insulin resistance.Insulin resistance decreased after treatment with ACT001 or PPC (Fig. 2C-D). These results confirm that, in mice fed a HFD, ACT001 can reduce insulin resistance and increase glucose tolerance.

### ACT001 can improve kidney damage in HFD-fed mice

**Fig. 3:**
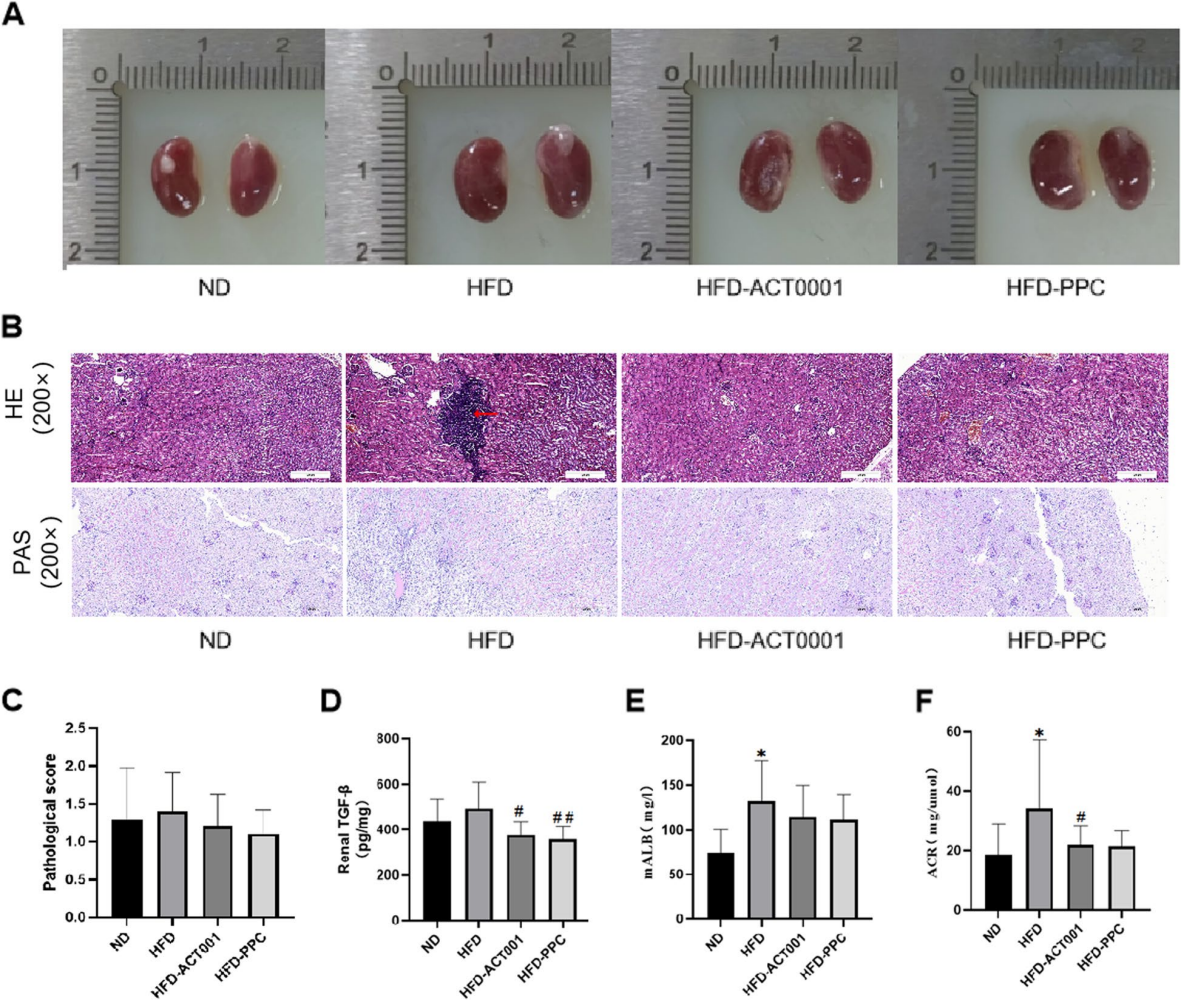
ACT001 can improve kidney damage in high-fat mice. (**A**) General picture of the kidney (**B**) HE staining of kidneys (200 ×) and (**C**) its pathological score (B) Renal PAS staining (200 ×) (**D**) Renal TGF-β (**E**) Urinary microalbumin (**F**) Urinary ACR *P < 0.05, **P < 0.01, ***P < 0.001, ****P < 0.0001 vs. ND; #*P* < 0.05, ##*P* < 0.01, ###*P* < 0.001, ####*P* < 0.0001 vs HFD

General renal observations revealed that the HFD group’s kidneys were larger than the ND mice’s kidneys, and the kidney volume decreased following treatment with ACT001 or PPC (Fig. 3A). HE staining of renal tissue revealed that the HFD mice display glomerular hypertrophy in renal tissue and infiltration of large lymphocytes in the local interstitium (Fig. 3B). PAS staining showed a rise in the glomerular mesangial matrix, a modest thickening in the glomerular capsule wall, and renal tubule atrophy. After treatment with ACT001 or PPC, there was some improvement in renal structure (Fig. 3B). Measurement of renal transforming growth factor-1 (TGF-β1) using ELISA showed that ACT001 alleviated renal fibrosis (Fig. 3D). Pathological scoring by HE staining showed that renal tissue morphology improved after treatment with ACT001 and PPC (Fig. 3C). The HFD mice had a significantly greater urinary microalbumin content than the ND mice. After treatment with ACT001 or PPC, urinary microalbumin decreased, and mice treated with PPC showed a more highly significant decrease (Fig. 3E).The albumin-to-creatinine ratio in the urine (ACR) is an important indicator in early kidney damage. The HFD mice had considerably greater urine ACR than the other groups. After ACT001 treatment, ACR decreased significantly. After PPC treatment, ACR decreased, but this decrease was not statistically significant (Fig. 3F). These results demonstrate that a HFD diet may result in alterations in renal tissue morphology, fibrosis and decreased renal filtration capacity. ACT001 can effectively alleviate renal injury.

### ACT001’s influence on gut metabolite SCFAs levels

**Fig. 4:**
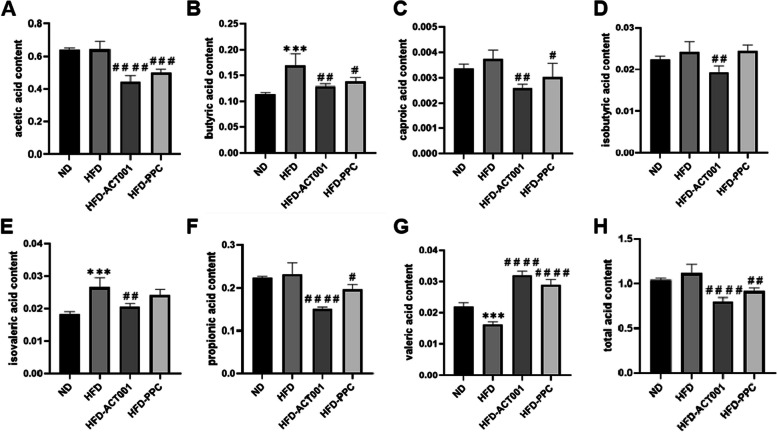
The effect of ACT001 on the content of the intestinal metabolite SCFA in high-fat mice. (**A**) acetic acid (**B**) butyric acid (**C**) caproic acid (**D**) isobutyric acid (**E**) isovaleric acid (**F**) propionic acid (**G**) valeric acid (**H**) total acid *P < 0.05, **P < 0.01, ***P < 0.001, ****P < 0.0001 vs. ND; #*P* < 0.05, ##*P* < 0.01, ###*P* < 0.001, ####*P* < 0.0001 vs HFD

To determine whether alterations in the gut microbiota after obesity affect the SCFAs levels in faeces, GC‒MS was used to analyse the relative levels and types of SCFAs in mouse faeces. High fat has little effect on acetic acid and propionic acid, but the levels decrease significantly after treatment (Fig. 4A and F). Butyric acid and isovaleric acid greatly rose in high-fat organs and notably decreased following ACT001 therapy (Fig. 4B and E). There was no statistically significant difference despite the fact that high fat led to a modest rise in caproic acid, isobutyric acid, and total acid. Following ACT001 therapy, there was a considerable drop (Fig. 4C, D and H). However, valeric acid levels dramatically rose following medication therapy, particularly ACT001, and significantly reduced in high-fat organs (Fig. 4G). Valeric acid may help lower obesity in high-fat animals, according to studies [21]. Therefore, by encouraging the formation of valeric acid, ACT001 could have a therapeutic impact.

### ACT001 reduces lipid buildup in the kidneys

**Fig. 5:**
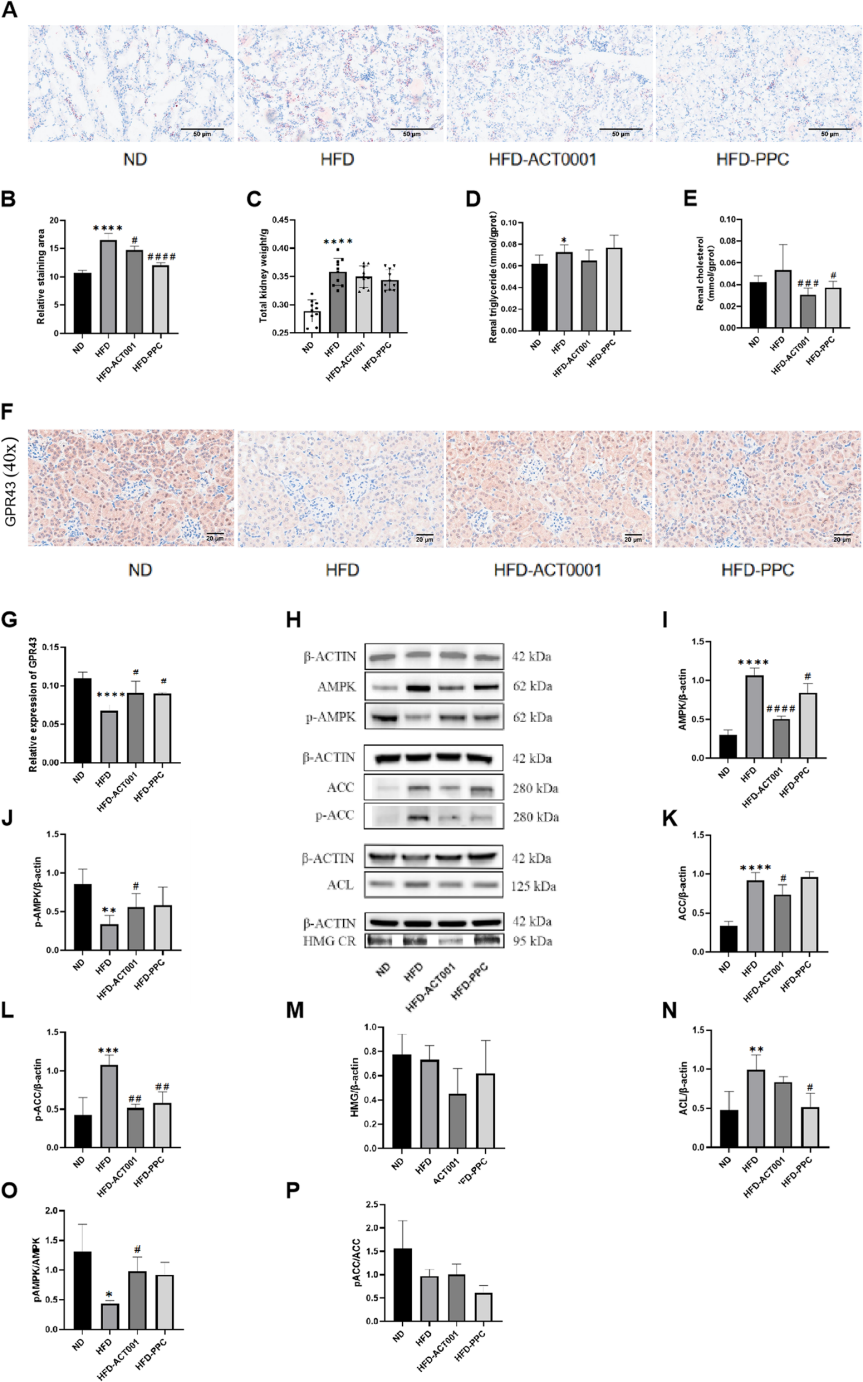
ACT001 alleviates renal lipid accumulation in hyperlipidemic mice. (**A**) Kidney oil red O staining and (**B**) its staining area (**C**) Kidney weight (**D**) kidney triglyceride content (**F**) GPR43 immunohistochemistry image (40 ×) (**G**) Quantitative analysis of GPR43 position areas (**H**) Western blot results of AMPK, p-AMPK, ACC, p-ACC, HMG CR, ACL (**I**) AMPK greyscale semi semi-quantitative analysis (**J**) p-AMPK greyscale semi-quantitative analysis (**K**) ACC greyscale semi-quantitative analysis (**L**) p-ACC greyscale semi-quantitative analysis (**M**) HMG CR greyscale semi-quantitative analysis (**N**) ACL greyscale semi-quantitative analysis (**O**) Pampk/AMPK (**P**) pACC/ACC

Oil red O staining of kidneys indicated an increase in the glomerular volume, mesangial cells and lipid deposition in HFD group compared to other groups. After drug therapy, lipid deposition in mouse kidney tissue decreased (Fig. 5A-B). The HFD-fed mice had considerably heavier kidneys than that in the ND group. After treatment with ACT001 or PPC, the kidney weight in the HFD mice decreased slightly, yet this distinction lacked statistical significance (Fig. 5C). The amount of renal triglycerides and cholesterol s were detected by ELISA. The HFD group displayed a greater triglyceride concentration than the ND group, yet no significant decrease was observed after ACT001 treatment.Cholesterol levels were increased in HFD mice but significantly decreased following ACT001/PPC treatment (Fig. 5D-E).

Indeed, a notable rise in renal GPR43 protein content was noticed treated with ACT001 or PPC compared to the HFD mice (Fig. 5F-G). Significantly increases in AMPK expression were detected in HFD than in ND mouse kidney, and these levels decreased significantly after ACT001 or PPC treatment. In contrast, p-AMPK expression levels significantly lower in HFD than in ND mouse kidney, yet these levels significantly rose following ACT001 or PPC treatment. The difference caused by ACT001 treatment was statistically significant (Fig. 5H-J). After normalization to beta-actin, the data were calculated and presented in terms of p-AMPK/AMPK ratios to assess the activated signalling pathways. The p-AMPK/AMPK levels in HFD mice were significantly lower than those in ND or ACT001 treatment mice, suggesting that ACT001 had a considerable effect on the AMPK pathway (Fig. 5O). The experimental results indicate that a HFD can induce total AMPK protein expression and that ACT001 or PPC treatment promotes AMPK phosphorylation.

The HFD mice displayed notably higher renal expression levels of p-ACC than the other groups, and after drug intervention, these levels were notably reduced–particularly in the HFD-ACT001 group (Fig. 5H, K, L). HFD mice had lower p-ACC/ACC than HFD-ACT001 mice (Fig. 5P). Activated AMPK phosphorylates ACC. This inactivates ACC and inhibits adipogenesis [22]. HFD significantly elevated the protein content of ATP citrate lyase (ACL) and 3-hydroxy-3-methylglutaryl CoA reductase (HMGCR) in mouse kidneys, and these levels significantly decreased after treatment with ACT001 or PPC (Fig. 5M-N). The above results indicate that the renal lipid synthesis pathway is activated in HFD-fed mice, and this leads to renal lipid accumulation. ACT001 may activate AMPK, encourage ACC phosphorylation, accelerate energy metabolism, and reduce lipid synthesis.

### ACT001 reduces kidney inflammation and oxidative damage

**Fig. 6:**
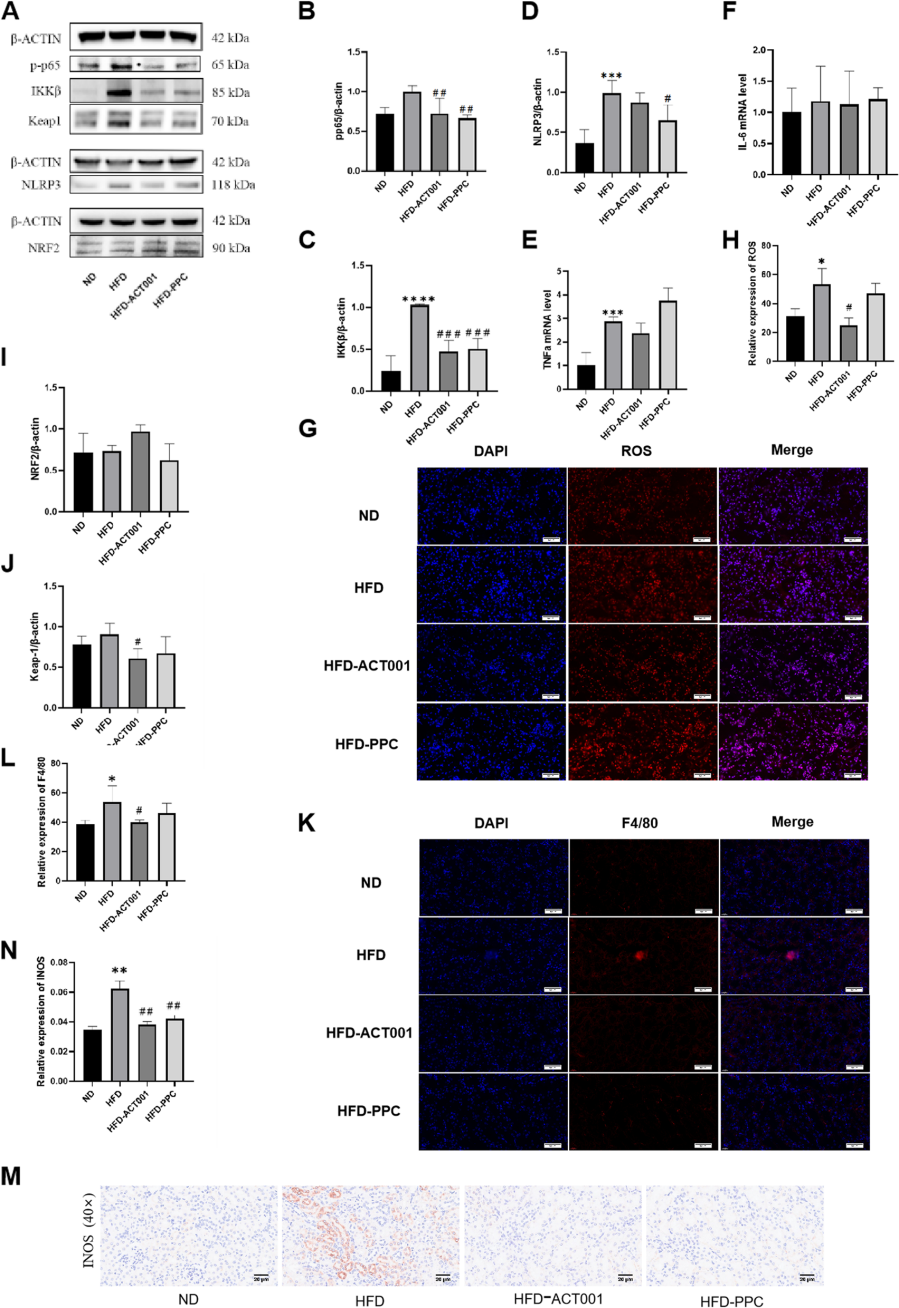
ACT001 reduce renal inflammation and oxide stress in ORG mice. (**A**) Western blot results of p-p65, IKKβ, NLRP3, NRF2, Keap 1 (**B**) p-p65 greyscale semi-quantitative analysis (**C**) IKKβgreyscale semi-quantitative analysis (**D** NLRP3 greyscale semi-quantitative analysis (**E**) TNF-a mRNA level (**F**) IL-6 mRNA level (**G**) ROS immunofluorescence image (40 ×) (H) ROS fluorescence intensity measurement (**I**) NRF2 greyscale semi-quantitative analysis (**J**) Keap1 semi-quantitative analysis (**K**) F4/80 immunofluorescence image (40 ×) (**L**) Quantitative measurement of F4/80 fluorescence intensity (**M**) INOS immunohistochemistry image (40 ×) (**N**) INOS positive regions quantitative analysis *P < 0.05, **P < 0.01, ***P < 0.001, ****P < 0.0001 vs. ND; #*P* < 0.05, ##*P* < 0.01, ###*P* < 0.001, ####*P* < 0.0001 vs HFD

HFD group levels of pp65, ikkβ, NLRP3, TNFa and IL6 were considerably greater than ND mice, drastically decreased following therapy with ACT001 or PPC. These findings indicate that ACT001 can downregulate the NF-κB pathway and inhibit inflammation (Fig. 6A-F).

Most studies suggest that NLRP3 activation is mainly related to oxygen species (ROS) production. HFD mice experienced a marked rise in ROS levels compare to ND mice, while ACT001 treatment had a significant effect on ROS levels (Fig. 6G and H). Furthermore, Nrf2 expression of protein and gene levels in HFD mouse kidneys were diminished, whereas Keap1 protein expression was augmented. After drug intervention, the content of Nrf2 were increased, whereas the content of Keap1 were decreased. These findings indicate that ACT001 and PPC have antioxidant defence effects against ORG and that ACT001 has better therapeutic effects (Fig. 6I and J).

Immunofluorescence staining was used to measure the level of F4/80 in mouse kidneys. More red signals on the cell membrane were found in the HFD mice, indicating strong expression of F4/80. Following ACT001 therapy, F4/80 expression was significantly reduced, and the positive rate of total macrophages was lower. The HFD-PPC group did not experience any significant changes (Fig. 6K and L). INOS content in mouse kidneys was measured by immunohistochemical staining. The HFD mice had greater INOS levels than the other groups. After drug treatment, INOS levels and M1-type macrophages were significantly reduced (Fig. 6M and N).

### The effect of ACT001 on the colonic barrier

**Fig. 7:**
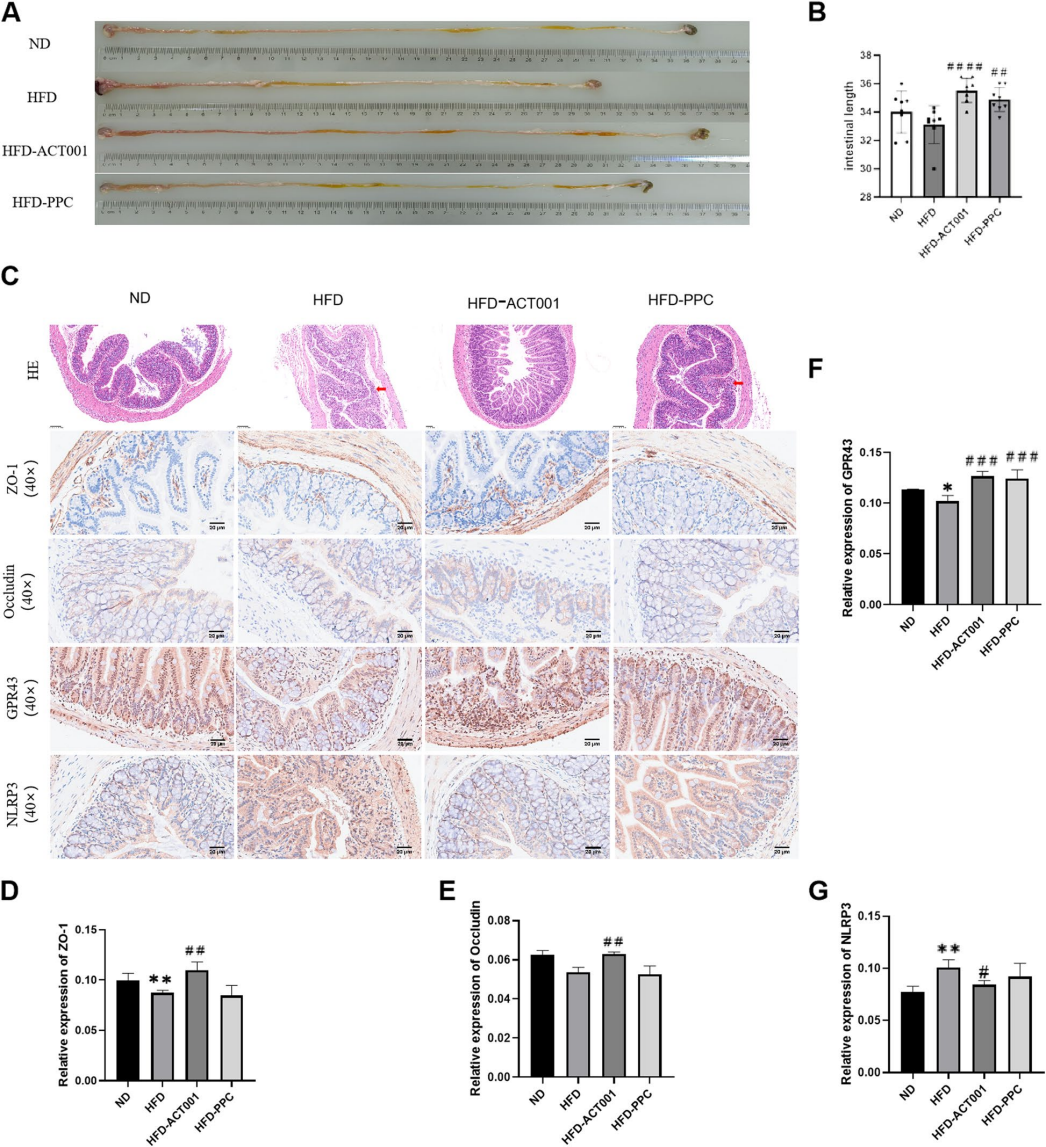
The effect of ACT001 on the colonic barrier. (**A**) Colon macroscopy (**B**) Colon length comparison (**C**) Colon HE staining (40 ×), immunohistochemical ZO-1 (40 ×), occluding (40 ×), GPR43 (40 ×), NLRP3 (40 ×) (D) ZO-1 positive area quantitative analysis (**E**) Occludin positive area *P < 0.05, **P < 0.01, ***P < 0.001, ****P < 0.0001 vs. ND; #*P* < 0.05, ##*P* < 0.01, ###*P* < 0.001, ####*P* < 0.0001 vs HFD

The intestinal length in HFD mice was substantially shorter than that in ND mice, and the colon's length was dramatically increased following treatment with ACT001 or PPC (Fig. 7A-B). The HE staining results showed that the HFD mice exhibited mild inflammatory reactions in the colon than that in the ND mice, with lesions invading the submucosa, and some crypts and epithelium were damaged. After ACT001 treatment, intestinal inflammation disappeared, and normal physiological intestinal structure was restored (Fig. 7C). Different types of epithelial cells and the complexes that link them physically constitute the intestinal barrier. As a result of the intestine's structural integrity, it guarantees physiological metabolism. Tightly associated complexes include claudin, ZO-1 and occludin [23]. A decrease in ZO-1 and occludin expression was observed due to an increase in intestinal permeability, a consequence of HFD [24]. However, this study revealed that ACT001 could significantly raise these levels in mice fed with HFDs, indicating it can stimulate TJs, thus sustaining intestinal mucosal barrier function and alleviating colitis (Fig. 7C and E). Through the GPR43 pathway, SCFAs can have an effect on the generation of inflammatory cytokines. This reduces chronic metabolic inflammation [25]. Immunohistochemical results revealed a marked decrease in the colonic expression of GPR43 in HFD mice, as opposed to ND mice, and an augmentation following ACT001 or PPC treatment (Fig. 7C and F). NLRP3 is essential for the formation of host anti-inflammatory and immune reactions [26]. SCFAs exert anti-inflammatory effects by binding to GPR43 to inhibit the activation of the NLRP3 inflammasome [27]. To determine whether the therapeutic effect of ACT001 involves NLRP3, we compared the colonic NLRP3 content of each group. The colonic NLRP3 content of HFD mice was markedly raised, while ACT001 treatment markedly lowered it, indicating that NLRP3 inflammasome activation can be prevented by ACT001 (Fig. 7C and G).

## Discussion

The current research aimed to elucidate the function of ACT001 in ORG. ACT001 can inhibit fibrosis and inflammation, defend against oxidative damage and regulate lipid metabolism to treat HFD-induced renal lipid accumulation and associated renal dysfunction and structural changes.

One of the major gut metabolites with anti-inflammatory properties are SCFAs. In the experiment, mice's intestinal SCFAs concentrations were measured.Reduced obesity traits in mice may be connected to increased valeric acid [21]. The sensor GPR43, which is mostly triggered by SCFAs, can control adipose tissue and maintain metabolic balance [28, 29]. Valeric acid has been discovered by scientists to activate GPR43 and control inflammation [30]. Mice were assessed for intestinal SCFA levels, and the amount of valeric acid was notably greater following ACT001 therapy compare to the HFD group. Renal GPR43 levels were significantly decreased in the HFD group, yet after ACT001 therapy, they rose again. By encouraging the generation of valeric acid and raising kidney GPR43, ACT001 may reduce obesity and inflammation.

GPR43 is bound and activated by SCFAs to induce AMPK phosphorylation [31]. As an energy sensor, AMPK regulates many enzymes involved in lipid homeostasis [32]. The phosphorylation of ACC is triggered by activated AMPK, thus diminishing the production of malonyl-CoA [33]. Moreover, Anne Emily et al. [34] found that AMPK activation can also inhibit cholesterol synthesis by regulating the rate-limiting cholesterol synthase HMGCR, which is consistent with this research. This indicate that ACT001 can inhibit ACL/HMGCR and alleviate lipid accumulation.

Widely accepted is the notion that chronic meta-inflammation brought on by obesity contributes to ORG developing [35]. Research has shown that the NF-ΚB signalling pathway enhances the obesity-induced inflammatory response in the kidney [36]. This research shows that the NF-κB pathway is activated in HFD-fed mice and that ACT001 can downregulate the NF-κB pathway, inhibiting the inflammatory reaction and delaying ORG development. Additionally, many studies have found that activating AMPK can block NF-κB, which is involved in regulating inflammatory signalling pathways [37]. Besides inflammation, AMPK and the profibrotic pathway are intimately related. Some researchers have found that AMPK activation can significantly reduce glomerular TGF-β in various diabetic nephropathy models [38]. Consistent with this, these research results show that the kidney TGF-β levels in HFD group mice are negatively correlated with the content of p-AMPK.

Macrophages are the major effector cells of kidney inflammation. Research has indicated that in the early stages of injury, macrophages can polarize into a proinflammatory phenotype, namely, M1-type cells, causing tissue or body damage. In the repair phase, macrophages adapt anti-inflammatory phenotypes, namely, M2-type cells, playing an anti-inflammatory role [39]. F4/80 is a macrophage marker that is intimately linked to the beginning and progression of inflammation and may accurately indicate the level of macrophage infiltration. Macrophages of M1 type generate INOS, which is a key inflammatory mediator, and also create considerable amounts of nitric oxide(NO)within the cell [40]. F4/80 and INOS expression were increased in HFD mice, indicating an increase in total macrophage infiltration and MI-type macrophage expression. However, these levels were decreased after treatment with ACT001. Speculation arose that ACT001 could either diminish M1-type macrophages or augment M2-type ones, thus curtailing inflammatory harm and postponing the advancement of fibrosis.

The second-largest oxygen-consuming organ in the human body is the kidney. To accommodate its high metabolic needs, it contains multiple mitochondria in each of its cells. Therefore, it can also produce reactive ROS and cause oxidative damage [34]. Nutrient excess from HFD leads to ectopic lipid buildup and the activation in renal prolipogenic and profibrotic genes. Increased oxidative damage and dysfunctional mitochondria are the consequences [41]. Mitochondrial dysfunction can worsen the ability to produce ATP, leading to an imbalance in cellular homeostasis and thus affecting organ function [42]. Oxidative stress is the main characteristic of CKD. Oxidative stress is usually linked to NF-κB activation. Inflammation and oxidative damage are mutually dependent processes that each contribute to and amplify [43]. The Nrf2/Keap1 signalling is initiated by an increasing ROS content, thereby reducing inflammation and oxidative stress [44]. Moreover, AMPK may have a positive effect on Keap1/Nrf2 signalling, thereby increasing cell defence against oxidative and other harmful damage [45]. This study showed that the antioxidant defence system in the HFD mice was damaged, and following ACT001 therapy, the Nrf2/Keap1 pathway was stimulated to activate antioxidant defence to avoid excessive ROS production.

Selective permeability of the intestinal physical barrier is mostly made up of tight junctions (TJs) and intestinal epithelial cells (IECs), with SCFAs being a key source of fuel for these cells. These substances are known to improve the functioning of the barrier and control its integrity by mediating TJs. The main components of TJs are claudin, occludin and ZO-1 [46]. SCFAs affect the intestinal tract through the GPR43 receptor, and mice exhibit worsening colitis after GPR43^−/−^ dextran sulfate sodium (DSS) injury [27]. The data demonstrate that ACT001 can promote the repair of intestinal tissue damage in ORG mice by binding SCFAs to GPR43, upregulating intestinal TJ proteins and resisting intestinal inflammation by inhibiting the NLRP3 inflammasome, thereby restoring the intestinal mucosal barrier.

### Strengths and limitations

Strength: A new sesquiterpene lactone derivative called ACT001 has both anti-inflammatory and antitumor properties. This is the first to investigate ACT001's possible treatment mechanism for ORG. It is anticipated that ACT001 will emerge as a novel medication candidate for the management of ORG.

Limitations: The sample size was not particularly great and only female C57BL/6 J mice were employed. Due to restrictions on study funding, only feces SCFAs were assessed, which do not give an accurate picture of mice's total metabolism. If the circumstances permit, spatial metabolomics can be employed in the future to analyze a variety of species and samples.

## Conclusion

**Fig. 8:**
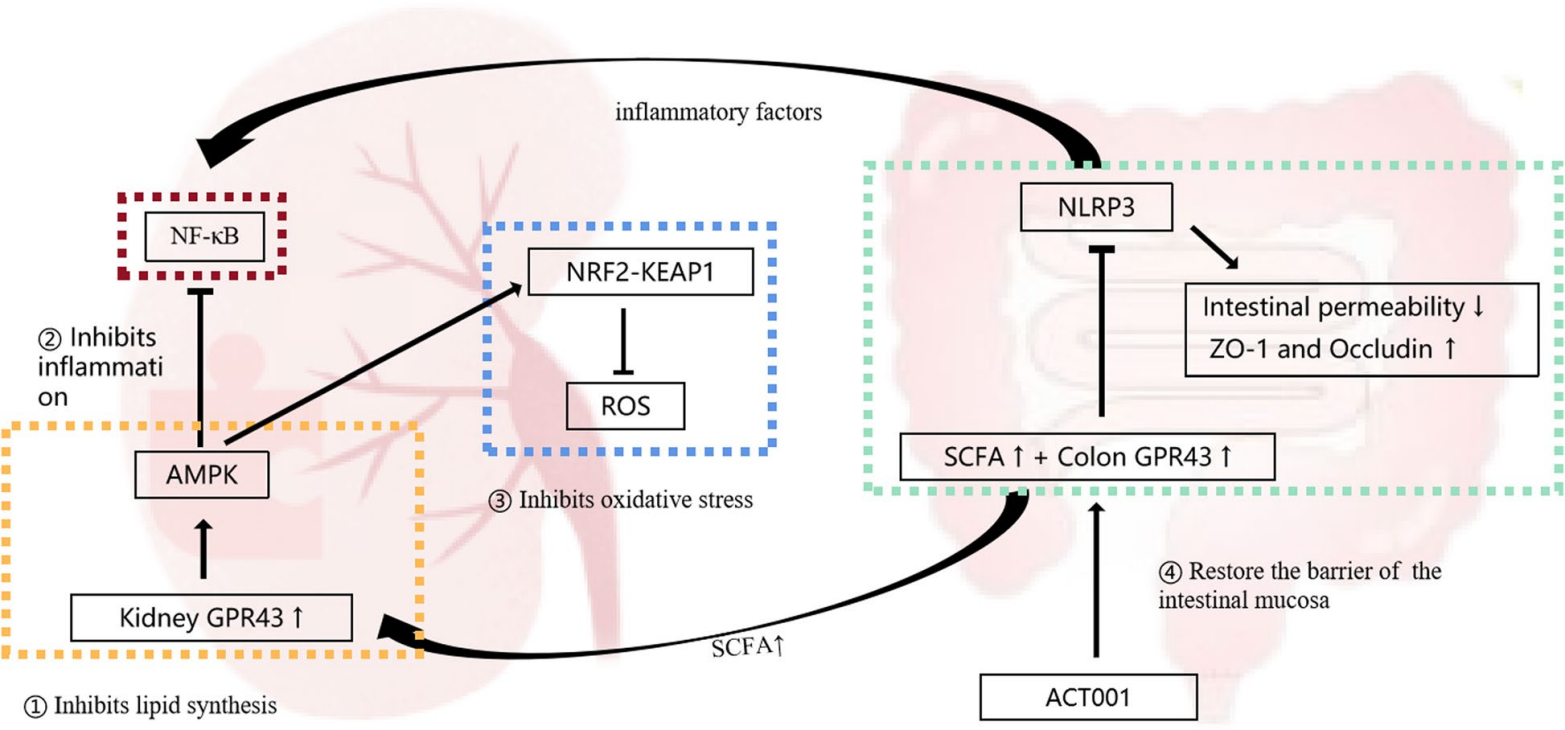
The therapeutic mechanism of ACT001 on ORG

In summary, ACT001 promotes the activation of colonic GPR43 by increasing the content of the SCFA valeric acid, restoring the colonic mucosal barrier, and preventing systemic inflammation while also increasing renal GPR43 levels, reducing kidney lipid buildup, and inhibiting inflammation and oxidative damage to achieve the therapeutic power of ORG (Fig. 8). Some people with ORG also need to take medication besides diet and exercise. However, there isn't a medicine that has been authorized to treat ORG. It is anticipated that ACT001 will emerge as a novel medication candidate for the management of ORG.

## Data Availability

Not applicable.

## Abbreviations

CKD: Chronic kidney disease
HFD: High-fat diet
ORG: Obesity-associated kidney disease
HE: Haematoxylin eosin
ESRD: End-stage renal disease
PAS: Periodic acid Schiff
SCFA: Short-chain fatty acid
FDA: Food and Drug Administration
IPGTT: Glucose tolerance test
PPC: Polyphosphocholine
ND: Normal diet
ACC: Acetyl-CoA carboxylase
LFD: Low-fat diet
AUC: Area under each blood glucose curve
HRP: Horseradish peroxidase
TNFa: Tumour necrosis factor a
IPITT: Insulin tolerance test
ANOVA: Univariate analysis of variance
IL6: Interleukin 6
ACL: ATP citrate lyase
ROS: Oxygen species
INOS: Inducible nitric oxide synthase
NO: Nitric oxide
IEC: Intestinal epithelial cells
HMGCR: 3-Hydroxy-3-methylglutaryl CoA reductase
TJ: Tight junction
